# ETV2 induces endothelial, but not hematopoietic, lineage specification in birds

**DOI:** 10.26508/lsa.202402694

**Published:** 2024-04-03

**Authors:** Wei Weng, Yuan Deng, Ruslan Deviatiiarov, Sofiane Hamidi, Eriko Kajikawa, Oleg Gusev, Hiroshi Kiyonari, Guojie Zhang, Guojun Sheng

**Affiliations:** 1 International Research Center for Medical Sciences, Kumamoto University , Kumamoto, Japan; 2 BGI Research, Wuhan, China; 3 Graduate School of Medicine, Juntendo University, Tokyo, Japan; 4 Institute of Fundamental Medicine and Biology, Kazan Federal University, Kazan, Russia; 5 RIKEN BDR, Kobe, Japan; 6 Life Improvement by Future Technologies (LIFT) Center, Moscow, Russia; 7 Department of Biology, University of Copenhagen, Copenhagen, Denmark; 8 Centre for Evolutionary & Organismal Biology, Zhejiang University, Hangzhou, China

## Abstract

Cardiovascular system develops from the lateral plate mesoderm. Its three primary cell lineages (hematopoietic, endothelial, and muscular) are specified by the sequential actions of conserved transcriptional factors. *ETV2*, a master regulator of mammalian hemangioblast development, however, is absent in the chicken genome and acts downstream of *NPAS4L* in zebrafish. Here, we investigated the epistatic relationship between NPAS4L and ETV2 in avian hemangioblast development. We showed that *ETV2* is deleted in all 363 avian genomes analyzed. Mouse ETV2 induced LMO2, but not NPAS4L or SCL, expression in chicken mesoderm. Squamate (lizards, geckos, and snakes) genomes contain both *NPAS4L* and *ETV2*. In Madagascar ground gecko, both genes were expressed in developing hemangioblasts. Gecko ETV2 induced only LMO2 in chicken mesoderm. We propose that both *NPAS4L* and *ETV2* were present in ancestral amniote, with ETV2 acting downstream of NPAS4L in endothelial lineage specification. ETV2 may have acted as a pioneer factor by promoting chromatin accessibility of endothelial-specific genes and, in parallel with *NPAS4L* loss in ancestral mammals, has gained similar function in regulating blood-specific genes.

## Abstract (precis)

ETV2, a master regulator of blood and vessel development in mammals, is deleted in bird genomes, and exogenous ETV2 induces endothelial lineage specification in nascent chicken mesoderm.

## Introduction

**Figure 1.**
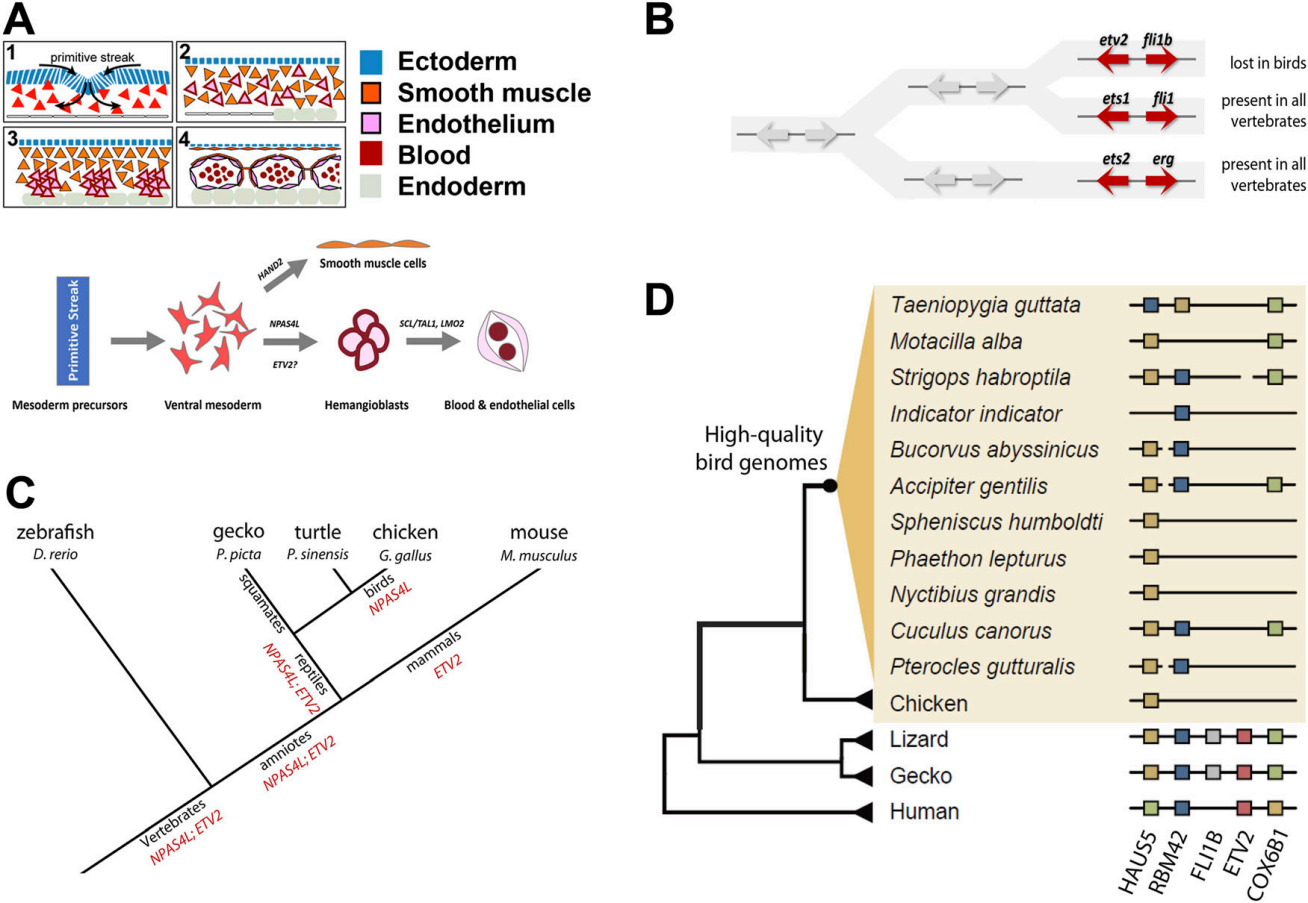
Hemangioblast specification and deletion of ETV2 locus in birds. **(A)** Top: Schematic diagram of ventral mesoderm morphogenesis, leading to the formation of its three main cell lineages: the blood, endothelium, and smooth muscle. Bottom: Three stages of hemangioblast development. Ventral mesoderm is derived from mesoderm precursors located in the primitive streak through epithelial–mesenchymal transition. Some ventral mesoderm cells are specified as smooth muscle progenitors and the remainder as hemangioblasts, possibly through the actions of master regulators such as NPAS4L and/or ETV2. Hemangioblasts express markers such as SCL/TAL1 and LMO2, and their differential actions lead to further specification as either blood or endothelial lineage. **(B)** There are three paralogous gene pairs in the vertebrate genome related to *ETV2*, likely as the result of whole genome duplication in early vertebrate evolution. *ETS1-FLI1* and *ETS2-ERG* pairs are conserved in all vertebrates. *ETV2-FLI1B* pair is deleted in chicken genome, and our current analysis reveals that this deletion is conserved in all birds. **(C)** A simplified view of vertebrate phylogenetic tree. Both *NPAS4L* and *ETV2* are hypothesized to be present in ancestral jawed vertebrates, ancestral amniotes, and ancestral reptiles. The clade leading to modern-day birds experienced gene loss of *ETV2*, and that leading to mammals experienced gene loss of *NPAS4L*. **(D)** The presence or absence of the *ETV2* and *FLI1B* genes in birds, reptiles, and humans through phylogenomic analysis. Species highlighted in yellow are the representative birds with high-quality genomes. Fragmented black lines indicate genes on different chromosomes/scaffolds. Squares show the presence of genes: *HAUS5*, green; *RBM42*, blue; *FLI1B*, grey; *ETV2*, red; and *COX6B1*, yellow. Deletion of *ETV2* and *FLI1B* is conserved in all birds. Deletion of neighboring genes (*HAUS5*, *RBM42*, and *COX6B1*) happened to variable degrees in different bird clades. Geckos and lizards have both *ETV2* and *FLI1B* in their genomes.

Vertebrate animals are built with a stereotypic body plan. Key developmental processes including gastrulation, germ layer patterning, cell lineage specification, and organ formation are under conserved morphogenetic and molecular regulation (Griffith & Wagner, 2017; Hu et al, 2017; Sheng et al, 2021; Steventon et al, 2021). We have previously reported that hematopoietic and vascular cell lineages in birds are derived from ventral mesoderm during gastrulation (Nakazawa et al, 2006; Alev et al, 2010) and that their differentiation is regulated by transcriptional factors and signaling pathways (Nakazawa et al, 2006; Shin et al, 2009; Weng & Sheng, 2014; Nagai et al, 2018) conserved in other vertebrates (Ciau-Uitz et al, 2014; Dzierzak & Bigas, 2018; Gore et al, 2018). The hematopoietic and vascular lineages are specified as common progenitors (called hemangioblasts) which subsequently give rise to either blood or endothelial cells (Choi et al, 1998; Huber et al, 2004; Vogeli et al, 2006; Weng et al, 2007; Nagai et al, 2018). Ventral mesoderm cells generate smooth muscle cells in addition to the hemangioblasts, and in birds, smooth muscle progenitors are segregated from hemangioblasts before the latter’s differentiation into either blood or endothelial lineage (Shin et al, 2009; Nagai et al, 2018) (Fig 1A).

Transcriptional factors regulating ventral mesoderm differentiation are also well-conserved. For example, in all vertebrate models studied, both SCL/TAL1 (Stem Cell Leukemia/T-cell Acute Lymphoblastic Leukemia 1) and LMO2 (LIM-Domain Only 2) mark nascent hemangioblasts (Gering et al, 2003; Jaffredo et al, 2005; Nakazawa et al, 2006; Patterson et al, 2007), and HAND2 (Heart and Neural Crest Derivatives Expressed 2) marks smooth muscle progenitors (Yamagishi et al, 2000; Shin et al, 2009; Skinner et al, 2010). Both SCL/TAL1 and LMO2 are involved in early hemangioblast development. SCL/TAL1 subsequently plays a more prominent role in promoting hematopoietic lineage specification (Porcher et al, 1996), and its expression becomes restricted to hematopoietic cells, whereas LMO2 is more prominently involved in endothelial differentiation (Yamada et al, 2000) with endothelium-restricted expression. Molecular conservation is further seen in other ventral mesoderm transcriptional regulators (e.g., GATA2 [GATA-binding Factor 2], GATA1 [GATA-binding Factor 1], RUNX1 [Runt-related Transcription Factor 1], and ETS1 [ETS Proto-Oncogene 1]) and in terminal differentiation markers (e.g., hemoglobin genes, CDH5 [Cadherin 5], ACTA2 [Actin Alpha 2]).

Timing of ventral mesoderm differentiation is strictly regulated in birds. SCL/TAL1 and LMO2 expression initiate at late Hamburger and Hamilton stage 4 (HH4) (Nakazawa et al, 2006; Weng et al, 2020), whereas ventral mesoderm formation starts from HH2 (about 8 h earlier) with the initiation of primitive streak formation and gastrulation epithelial–mesenchymal transition (Nakaya et al, 2008, 2013; Hamidi et al, 2020). Hematopoietic lineage terminal differentiation starts from HH7, about 8 h after expression of SCL/TAL and LMO2, with the onset of embryonic hemoglobin expression (rho, epsilon, and pi globins) (Nakazawa et al, 2006). The delay between mesoderm formation and SCL/TAL and LMO2 expression suggested that additional genes may act as hemangioblast master regulators.

One such candidate gene is ETS Variant Transcription Factor 2 (*ETV2*). In mice, *ETV2* functions before hemangioblast specification, and its knockout leads to loss of both hematopoietic and endothelial lineages (Kataoka et al, 2011; Liu et al, 2015). In zebrafish, however, *ETSRP* (ETS1 Related Protein; the ETV2 ortholog in fish) (Sumanas & Lin, 2006) functions under the control of another gene, *NPAS4L* (*cloche*) (Reischauer et al, 2016; Marass et al, 2019), which is viewed as bona fide hemangioblast master regulator (Stainier et al, 1995; Liao et al, 1998; Reischauer et al, 2016; Marass et al, 2019). In chickens, ortholog for *NPAS4L*, but not *ETV2*, has been identified (Weng et al, 2020), suggesting that *ETV2* is not necessary for hemangioblast development in birds and arguing against ETV2’s conserved role as a hemangioblast master regulator in amniotic vertebrates (mammals, birds, and reptiles). Interestingly, *NPAS4L* ortholog has not been reported in any mammal genome either, and a paralogous gene, *NPAS4*, does not appear to play any role in normal hemangioblast development (Lin et al, 2008; Spiegel et al, 2014). These lines of evidence suggest that neither ETV2 nor NPAS4L can be viewed as a conserved hemangioblast master regulator in vertebrates.

To understand how evolutionarily conserved developmental processes and cell lineages can be under the control of transcription factors that are functionally and phylogenetically divergent, we investigated the genomic organization of the *ETV2* gene in Aves and Reptilia and analyzed the functional relationship between ETV2, NPAS4L, SCL/TAL1, and LMO2.

## Results and Discussion

### *ETV2* gene is deleted in aves

ETV2 is essential for hemangioblast development in mice and important for vascular and myeloid development in zebrafish (Sumanas & Lin, 2006; Sumanas et al, 2008; Kataoka et al, 2011; Liu et al, 2015). In the chicken genome, we were unable to find the *ETV2* ortholog (Weng et al, 2020). To clarify when this gene may have been lost during avian evolution, we performed phylogenomic analysis of the *ETV2* gene in birds and non-avian reptiles. ETV2 belongs to the ETS family of transcription factors, the first member of which was chicken ETS1, a cellular counterpart of v-ets oncogene in avian leukemia retrovirus E26 (Leprince et al, 1983; Sizemore et al, 2017). Among 12 subfamilies of ETS-domain proteins (Laudet et al, 1999), ETV2 is a member of the ER71 subfamily (ETV2 is also called ER71 or ETSRP), closely related to another subfamily (the ETS subfamily) that includes ETS1 and ETS2 genes. Our analysis revealed that all three genes, *ETV2*, *ETS1*, and *ETS2*, were paralogous to each other, likely formed as a result of genome duplications in ancestral vertebrates (Lautenberger et al, 1992; Garrett-Sinha, 2013; Yu et al, 2023), and that each had a neighboring ETS family gene belonging to the ERG subfamily, forming three tandemly duplicated ETS gene pairs (*ETS1–FLI1*; *ETS2–ERG*; *ETV2–FLI1B*) (Fig 1B). Both the *ETS1–FLI1* and *ETS2–ERG* pairs are conserved, whereas the *ETV2–FLI1B* pair and its neighboring genes are deleted in the chicken genome. After extensive search using newly generated avian genome data from the B10K initiative (363 species representing 218 bird families) (Feng et al, 2020) (see the Materials and Methods section), we found that loss of *ETV2–FLI1B* gene pair was conserved in all avian species analyzed (Fig 1B–D), strongly suggesting a deletion event predating the appearance of modern birds. Syntenic analysis revealed that neighboring genes *COX6B1*, *RBM42*, and *HAUS5* also exhibited various degrees of lineage-specific loss in bird genomes (Fig 1D).

The squamates (a group of non-avian reptiles including modern-day lizards and snakes) retained both the *ETV2* and *FLI1B* genes and the neighboring *COX6B1*, *RBM42*, and *HAUS5* genes (Fig 1C and D). Mammalian genomes lost the *FLI1B* gene when retaining the three neighboring genes (Fig 1D). These data suggested that ancestral amniotes had both *ETV2* and *FLI1B* genes in a genomic locus linked to *COX6B1*, *RBM42*, and *HAUS5* genes, with subsequent loss of *ETV2* and *FLI1B* in birds (Fig 1D), of *FLIB* in mammals (Fig 1D), and of *ETV2* in turtles (UCSC genome browser gateway; data not shown). Linkage of *ETV2* gene to *RBM42* and *HAUS5* genes was seen in amphibia (Tibetan frog, *X. levis*, and *X. tropicalis*) and to *RBM42* gene in teleost fish (zebrafish, medaka, and tetraodon) (UCSC genome browser gateway), suggesting that syntenic organization of *ETV2* and its neighboring genes in ancestral amniotes was the result of rearrangements of homologous syntenic blocks during tetrapod evolution (Sacerdot et al, 2018; Damas et al, 2021, 2022).

### Avian NPAS4L marks mesoderm progenitors that will give rise to the blood and endothelium, but not the smooth muscle

**Figure 2.**
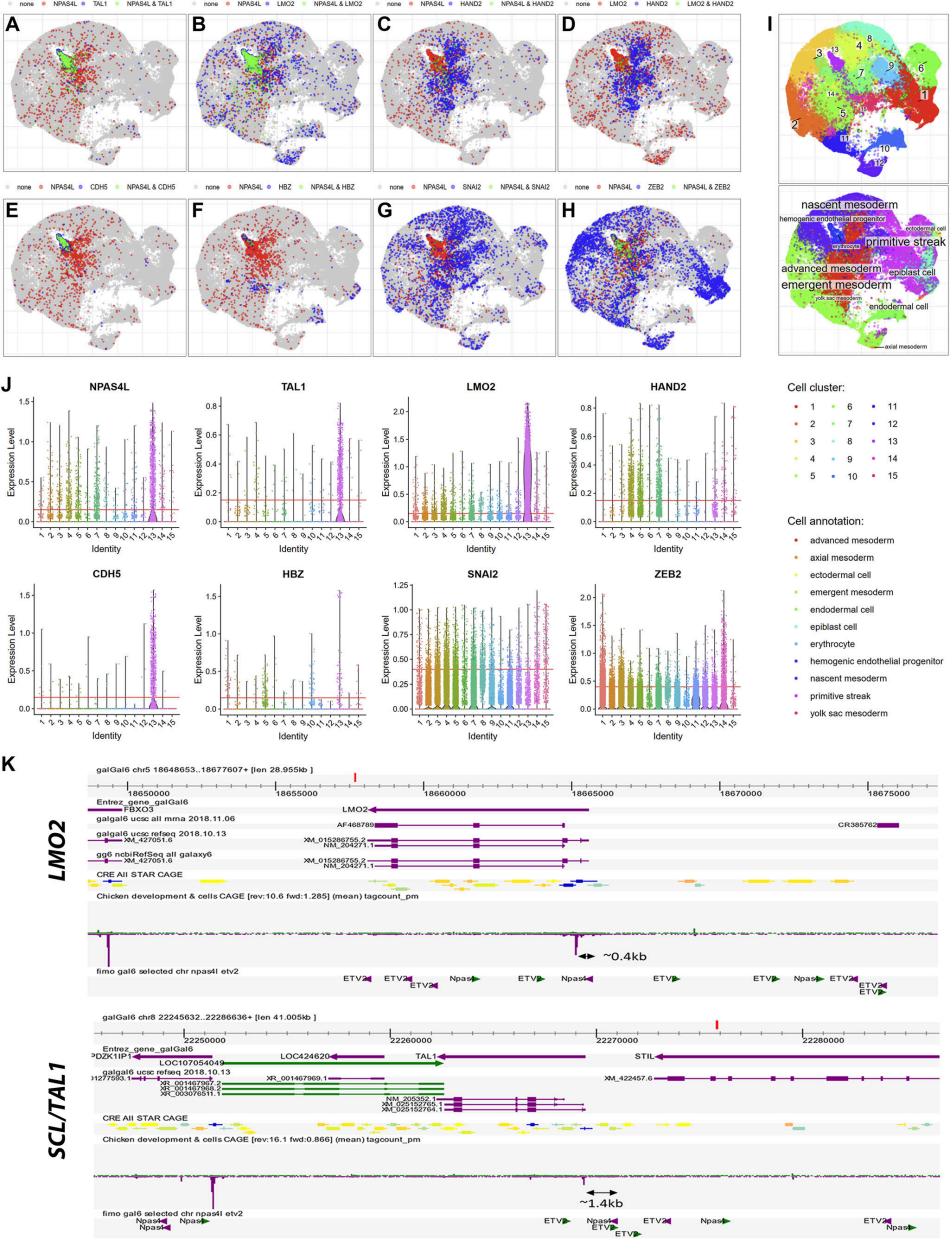
scRNAseq profiles of selected hemangioblast-related genes in chicken embryo (HH4-11) and binding sites for NAPAS4L and ETV2 in chicken SCL/TAL1 and LMO2 loci. **(A, B, C, D, E, F, G, H)** Gene expression overlaps for selected gene pairs. **(J)** Cells are colored in case if expression level passes thresholds defined in panel (J); green for double-positive cells; grey if expression is below threshold for both genes. **(A)** NPAS4L-SCL/TAL1. **(B)** NPAS4L-LMO2. **(C)** NPAS4L-HAND2. **(D)** LMO2-HAND2. **(E)** NPAS4L-CDH5. **(F)** NPAS4L-HBZ. **(G)** NPAS4L-SNAI2. **(H)** NPAS4L-ZEB2. Note: NPAS4L is annotated as NPAS4, incorrectly, in chicken genome. **(J)** The correct name is used here. Cells are colored if expression level passes thresholds (see (J)). Green for double-positive cells; grey if expression is below threshold for both genes. **(I)** Integrated single-cell datasets with clusters and inferred annotation. **(J)** Expression profiles and threshold selection for selected genes to reduce redundancy (SNAI2, ZEB2) or for non-specific expression (all the rest genes). See the Materials and Methods section for raw dataset description. **(K)** Optimal binding sites for NPAS4L and ETV2 are enriched in chicken *SCL/TAL1* and *LMO2* loci. See text for consensus binding sites of NPAS4L and ETV2. Chicken *SCL/TAL1* and *LMO2* loci are viewed using ZENBU browser and galGal6 assembly. Binding sites are located both in the proximity of transcription start site for both genes and in the gene body (for *LMO2*) and in the vicinity of gene locus (for both).

Lack of *ETV2* in avian genomes suggested that, unlike in mammals, another transcription factor may function as a hemangioblast master regulator in birds. RNA in situ hybridization analysis showed that endogenous chicken *NPAS4L* was expressed transitorily in hemangioblasts and that ectopic activation of either chicken or zebrafish NPAS4L was able to induce both SCL/TAL1 and LMO2, two conserved hemangioblast markers (Weng et al, 2020). At the single cell level, RNAseq analysis (HH4–HH11) (see the Materials and Methods section) (Williams et al, 2022; Rito et al, 2023 *Preprint*) revealed that NPAS4L+ cells were co-positive for SCL/TAL1 (Fig 2A, I, and J) and LMO2 (Fig 2B, I, and J) and were mutually exclusive with HAND2+ smooth muscle progenitor cells (Fig 2C, D, I, and J), in agreement with our whole-mount expression and functional analysis data (Shin et al, 2009). Because NPAS4L marks the early phase of hemangioblast development, at later stages, SCL/TAL1 and LMO2-positive cells became negative for NPAS4L, and only a very small percentage of NPAS4L-positive cells co-expressed terminal differentiation markers for endothelium (CDH5) (Fig 2E, I, and J) and blood (HBZ, hemoglobin pi) (Fig 2F, I, and J). SNAI2 (Fig 2G, I, and J) and ZEB2 (Fig 2H, I, and J), two EMT (epithelial mesenchymal transition) transcription factors expressed during ventral mesoderm differentiation, exhibited different and dynamic co-expression patterns with NPAS4L, suggesting that nascent ventral mesoderm cells are under complex morphological and migratory regulation in addition to cell fate specification (see later part of this work).

### Exogenously expressed mouse ETV2 up-regulates avian LMO2, but not SCL/TAL1 expression

Mouse ETV2 was shown to regulate both erythropoietic and endothelial gene regulatory networks, and its knockout affected the development of both lineages (Kataoka et al, 2011; Sumanas & Choi, 2016; Koyano-Nakagawa & Garry, 2017; Sinha et al, 2022). Coding and noncoding sequences regulating a given developmental trait, however, may follow separate evolutionary trajectories (e.g., in genes and regulatory sequences controlling mammalian hairlessness) (Kowalczyk et al, 2022). If deletion of avian *ETV2* gene was a recent event, its target genes may still retain cis-regulatory elements reflecting its ancestral roles in erythropoietic and endothelial lineage specification. Indeed, optimal binding sites for both ETV2 (Shrestha et al, 2022) and NPAS4L (Marass et al, 2019) were detected in chicken *SCL/TAL1* and *LMO2* loci (Fig 2K).

**Figure 3.**
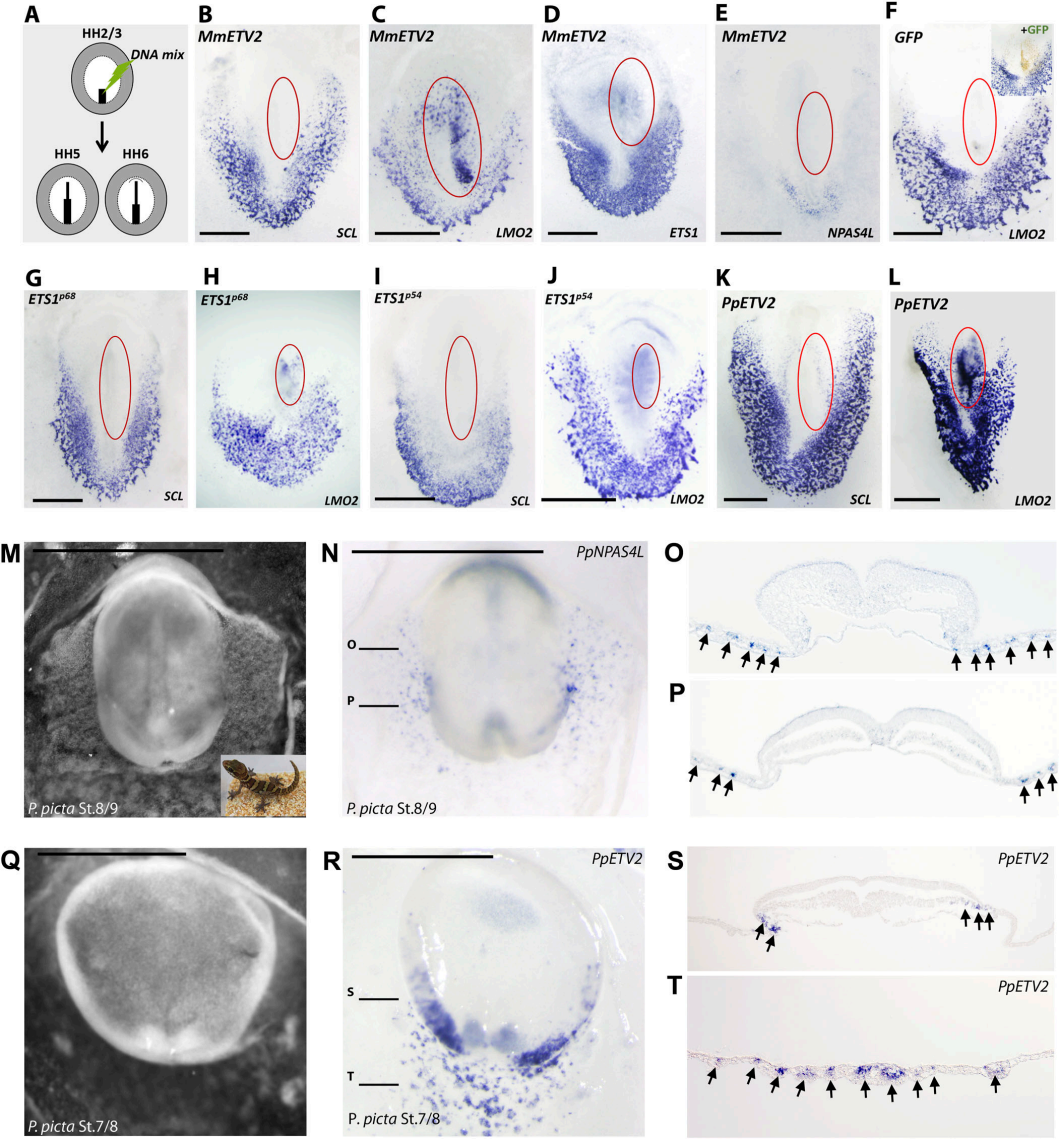
ETV2 induces LMO2, but not SCL/TAL1 expression in chicken mesoderm. **(A)** Schematic diagram of introducing ETV2 expression construct by electroporation at HH2/3 and analyzing ectopic expression of either SCL/TAL1 or LMO2 at HH5/6. **(B, C, D, E, F, G, H, I, J, K, L)** Blue: RNA expression of endogenous genes related to hemangioblast development. **(B, C, D, E, F, G, H, I, J, K, L)** Red oval: Area of exogenous gene expression based on GFP fluorescence after culture (MmETV2 in (B, C, D, E); control in (F); ETS1 in (G, H, I, J); PpETV2 in (K, L)). **(B)** MmETV2 does not induce endogenous SCL/TAL1 expression. **(C)** MmETV2 induces endogenous LMO2 expression strongly. **(D)** MmETV2 does not induce endogenous ETS1 expression. **(E)** MmETV2 does not induce endogenous NPAS4L expression. **(F)** Control GFP does not induce LMO2 expression. **(G)** ETS1-p68 does not induce endogenous SCL/TAL1 expression. **(H)** ETS1-p68 induces LMO2 expression very weakly. **(I)** ETS1-p54 does not induce SCL/TAL1 expression. **(J)** ETS1-p54 induces LMO2 expression very weakly. **(K)** PpETV2 does not induce SCL/TAL1 expression. **(L)** PpETV2 induces LMO2 expression strongly. **(M)** Dark-field view of *P. picta* embryo (St. 8/9) stained for PpNPAS4L expression. Inset: Image of an adult *P. picta*. **(N)** Bright-field view of *P. picta* embryo (St. 8/9) stained for PpNPAS4L expression. **(O, P)** Section levels shown in (O, P) are indicated by black lines. **(N, O, P)** Section of embryo shown in (N). Arrows indicate positive PpNPAS4L staining in hemangioblasts. **(Q)** Dark-field view of *P. picta* embryo stained for PpETV2. **(R)** Bright-field view of *P. picta* embryo stained for PpETV2. **(S, T)** Section levels shown in (S, T) are indicated by black lines. **(R, S, T)** Sections of embryo are shown in (R). Arrows indicate positive PpNPAS4L staining in hemangioblasts. Scale bar: 1 mm.

To investigate whether these binding sites reflected bona fide direct transcriptional regulation as was reported for ETV2 during mouse hemangioblast differentiation (Wareing et al, 2012), we cloned full-length mouse ETV2 cDNA (NM_007959) (see the Materials and Methods section) into the pCAGGS-2A-eGFP expression vector (Weng & Sheng, 2014) and tested its ability to induce blood (SCL/TAL1+) and/or endothelial (LMO2+) lineage differentiation in chicken mesoderm. The construct contained an eGFP-encoding gene separated from ETV2 by a 2A-peptide sequence (Weng & Sheng, 2014), and electroporated cells were identified by their GFP-positive signals. Early primitive streak stage (HH2-3) chicken embryos were electroporated with either control (GFP-only) or ETV2-expressing construct and analyzed for SCL/TAL1 and LMO2 expression at HH5-6, when blood and endothelial lineages started to be specified (Fig 3A), and lineage-specific segregation of SCL/TAL1-positive (in blood) and LMO2-positive (in endothelium) cells was initiated. Control construct did not induce either SCL/TAL1 or LMO2 expression as we had previously reported (Weng & Sheng, 2014; Weng et al, 2020) (in this experiment, 0/5 for SCL/TAL1; 0/6 for LMO2) (Fig 3F). Mouse ETV2 was able to induce LMO2 expression strongly (Fig 3C) (10/12), but not SCL/TAL1 (Fig 3B) (0/9) or the hemangioblast marker NPAS4L (Fig 3E) (0/9), suggesting that exogenously expressed ETV2 could still induce endothelial lineage specification despite its deletion in all bird genomes.

### Avian ETS1 does not compensate the putative hemangioblast-inducing function of ETV2

As normal chicken hemangioblast development does not require ETV2, we asked whether its function might have been replaced by another ETS-domain protein. Of 22 ETS-domain-containing genes found in the chicken genome (*ETS1*, *ETS2*, *ELF1*, *ELF2*, *ELF3*, *ELF5*, *ETV1*, *ETV3*, *ETV3L*, *ETV4*, *ETV5*, *ETV6*, *ETV7*, *EHF*, *ELK3*, *ELK4*, *FLI1*, *ERG*, *FEV*, *SPDEF*, *SPI1*, and *SPIC*) (GRCg6a; ensembl.org), 12 (*ETS1*, *ETS2*, *ELF1*, *ELF2*, *ELF5*, *ETV1*, *ETV3*, *ETV5*, *ETV6*, *ELK3*, *ELK4*, and *FLI1*) were shown to be expressed at stage HH3-8 (stages spanning hemangioblast formation and early differentiation) based on chicken developmental promoterome data we had published previously (Lizio et al, 2017). None of these genes matched temporal expression profiles of known hemangioblast markers (SCL/TAL1, LMO2, or NPAS4L) (Nakazawa et al, 2006; Shin et al, 2009; Weng et al, 2020). Two of them (ETS1 and FLI1) exhibited temporal patterns suggestive of their potential involvement in endothelial, but not blood, development (Nakazawa et al, 2006) (http://geisha.arizona.edu/).

In mice, loss of function of *ETS1* or *FLI1* gene did not affect hemangioblast development but rather resulted in vascular integrity and late-stage hematopoietic lineage differentiation phenotypes (Bories et al, 1995; Spyropoulos et al, 2000; Gao et al, 2010; Li et al, 2015). Although sharing low sequence homology outside the ETS domain, *ETS1* is paralogous to *ETV2*. We asked whether ETS1 could be functioning like ETV2 in chicken hemangioblast development. Exogenous ETV2 did not induce endogenous ETS1 expression (Fig 3D) (0/3). We cloned both versions of chicken ETS1 (ETS1-p68 and ETS1-p54) (Crepieux et al, 1993) into pCAGGS-2A-eGFP vector and analyzed their ability to induce SCL/TAL1 or LMO2 gene. Chicken ETS1 was unable to induce SCL/TAL1 (0/5 for ETS1-p68 and 0/5 for ETS1-p54) (Fig 3G and I) and only induced LMO2 sporadically (1/6 for ETS1-p68 and 1/6 for ETS1-p54) (Fig 3H and J). Similar results were observed when we used an alternative method (CRISPRon) (Weng et al, 2020) to turn on endogenous ETS1 transcription (0/4 for SCL/TAL1, 1/13 for LMO2), suggesting that chicken EST1 could not perform the putative role of ETV2 as a hemangioblast regulator. These data showed that mouse ETV2 was able to induce endothelial, but not hematopoietic, differentiation in chicken mesoderm and that neither ETV2 nor its paralog ETS1 met the criterion of a master regulator of hemangioblast specification.

### Squamate genome contains both *ETV2* and *NPAS4L*, and both genes are expressed in putative hemangioblast cells during early development in Madagascar ground gecko

These observations suggested that, in stem amniote, ETV2 may have functioned primarily as an endothelial inducer instead of hemangioblast inducer. However, it may also be possible that *ETV2* gene has evolved in its DNA and cofactor binding specificities so that mouse *ETV2* could not regulate hematopoietic gene expression in birds. To test this, we decided to clone *ETV2* gene from a non-avian reptile species. Our comparative phylogenomic analysis (Fig 1) revealed that both *NPAS4L* and *ETV2* genes are retained in green anole, Madagascar ground gecko, and python, suggesting that squamates (lizards and snakes) resemble anamniotic vertebrates (e.g., amphibians and teleosts) in having both the *NPAS4L* and *ETV2* genes in their genomes. Madagascar ground gecko (*Paroedora picta*) was recently shown to be a tractable animal model for evolutionary developmental studies (Hara et al, 2015, 2018; Yoshida et al, 2016; Kajikawa et al, 2020), and its developmental staging system has been well documented (Noro et al, 2009; Yoshida et al, 2016). We decided to investigate ETV2 and NPAS4L expression in Madagascar ground gecko embryos.

Pre-ovipositional (before egg-laying) development of *P. picta* takes ∼10 d, and freshly laid eggs are at a developmental stage equivalent to HH11-12 chicken embryos, too late for hemangioblast specification study. We therefore retrieved pre-ovipositional embryos from gecko oviducts, and embryos equivalent to HH5-7 (gecko Stage 7–9; [Yoshida et al, 2016; Kajikawa et al, 2020]) were used for RNA in situ hybridization analysis, using the same protocol as reported for chicken embryos (Alev et al, 2013). *P. picta* *NPAS4L* and *ETV2* genes (see the Materials and Methods section) were amplified from the cDNA prepared from pre-ovipositional stage *P. picta* embryos. RNA whole-mount in situ hybridization study showed that both gecko *NPAS4L* (*PpNPS4L*) (Fig 3M and N) and *ETV2* (*PpETV2*) (Fig 3Q and R) genes were expressed in the lateral plate/extraembryonic territories. Paraffin-sections of stained embryos revealed hemangioblast-specific staining of both genes (Fig 3O and P for PpNPAS4L and Fig 3S and T for PpETV2). Sequence comparison revealed that *ppETV2* was most closely related to amphibian (*X. tropicalis*) *ETV2* (84.7% amino acid sequence identity in the ETS domain; 34.2% amino acid sequence identity in the full length), followed by teleost (*D. rerio*) *ETV2* (76.2% and 32.3%, respectively) and mammalian (*M. musculus*) *ETV2* (63.9% and 28.8%, respectively). These data suggested that in squamates, both *NPAS4L* and *ETV2* genes are expressed at stages and in cell lineages supportive of their potential involvement in hemangioblast specification in stem amniotes.

### Gecko ETV2 induces LMO2, but not SCL/LMO2 expression in chicken mesoderm

Gecko ETV2 likely resembles ETV2 in ancestral sauropsids in both its protein sequence and molecular functions. We then asked whether gecko ETV2 was capable of regulating both hematopoietic and endothelial development, as its expression pattern would suggest. Manipulation of post-ovipositional gecko embryos has been reported (Nomura et al, 2015). However, molecular perturbation before egg-laying is still impractical in any reptilian embryo. We therefore cloned gecko ETV2 in pCAGGS-2A-eGFP expression construct (see the Materials and Methods section) and tested its ability to induce SCL/TAL1 and LMO2 in chicken mesoderm. Similar to mouse ETV2, gecko ETV2 induced ectopic LMO2 expression strongly (Fig 3L) (5/6), but failed to induce SCL/TAL1 (Fig 3K) (0/5) or NPAS4L (0/6).

### EMT transcription factor SNAI2 does not interfere with hemangioblast lineage specification

**Figure 4.**
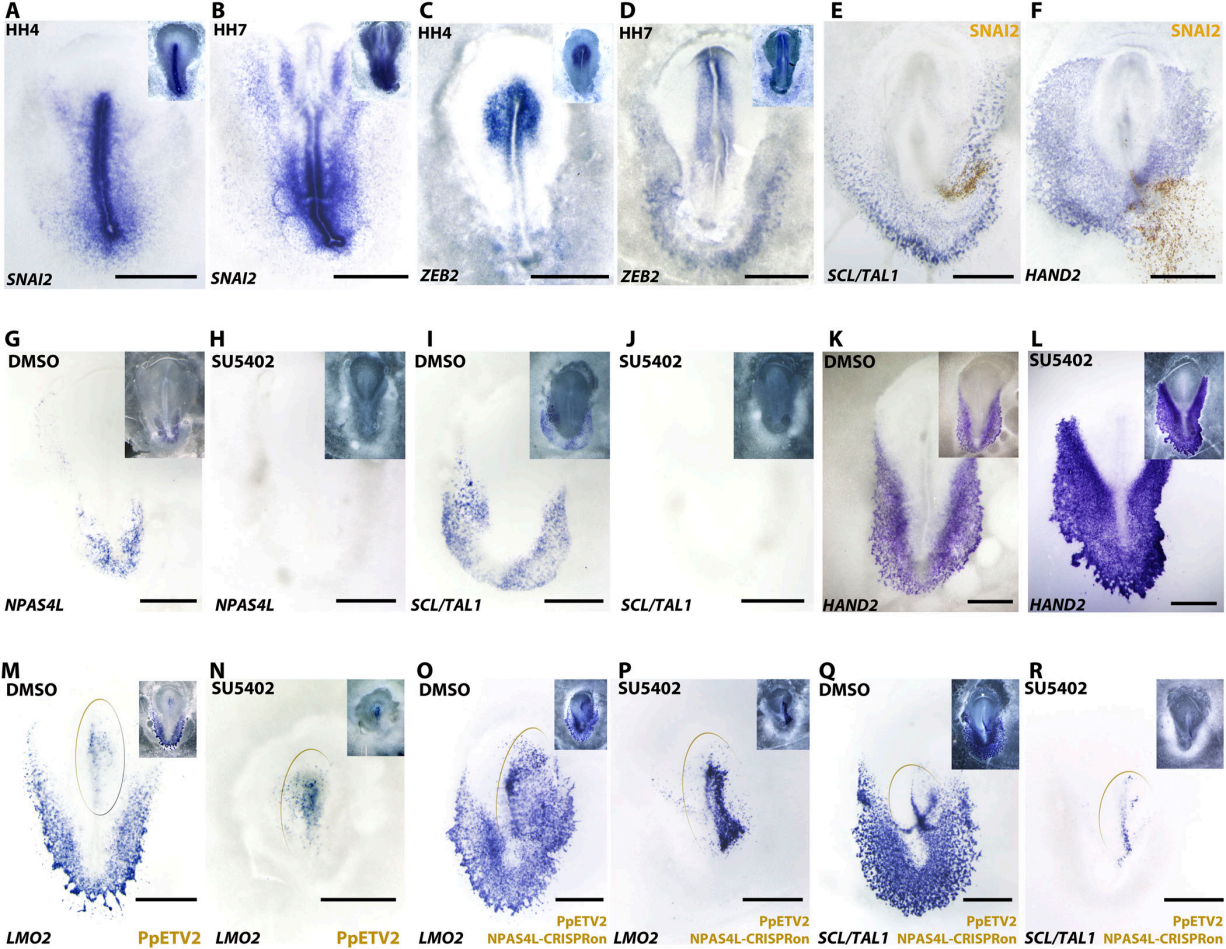
Effect of EMT regulator overexpression and receptor tyrosine kinase inhibition on hemangioblast specification. **(A, B, C, D)** Normal expression of EMT transcription factors SNAI2 and ZEB2 during hemangioblast development. Inset: dark-field view showing embryo stage more clearly. **(A)** SNAI2 at HH4. **(B)** SNAI2 at HH7. **(C)** ZEB2 at HH4. **(D)** ZEB2 at HH7. **(E)** Ectopic expression of SNAI2 does not affect endogenous SCL/TAL1 expression. **(F)** Ectopic expression of SNAI2 does not affect endogenous HAND2 expression. **(G, H, I, J, K, L, M, N, O, P, Q, R)** Effect of DMSO or SU5402 treatment on hemangioblast development. In (M, N, O, P, Q, R), territories of over-overexpression are marked by brown ovals. **(G)** Control DMSO treatment does not affect endogenous NPAS4L expression. **(H)** SU5402 treatment abolishes endogenous NPAS4L expression. **(I)** Control DMSO treatment does not affect endogenous SCL/TAL1 expression. **(J)** SU5402 treatment abolishes endogenous SCL/TAL1 expression. **(K)** Control DMSO treatment does not affect endogenous HAND2 expression. **(L)** SU5402 treatment increases endogenous HAND2 expression. **(M)** DMSO treatment does not affect induction of endogenous LMO2 expression by exogenous PpETV2. **(N)** SU5402 treatment does not affect induction of endogenous LMO2 expression by exogenous PpETV2. Note that SU5402 abolishes all endogenous LMO2 expression outside the exogenous PpETV2 territory. **(O)** DMSO treatment does not affect induction of endogenous LMO2 by a combination of exogenous PpETV2 and CRISPRon-mediated endogenous NPAS4L. **(P)** SU5402 treatment does not affect induction of endogenous LMO2 by a combination of exogenous PpETV2 and CRISPRon-mediated endogenous NPAS4L. Note that SU5402 abolishes all endogenous LMO2 expression outside the exogenous overexpression territory. **(Q)** DMSO treatment does not affect induction of endogenous SCL/TAL1 by a combination of exogenous PpETV2 and CRISPRon-mediated endogenous NPAS4L. **(R)** SU5402 treatment does not affect induction of endogenous SCL/TAL1 by a combination of exogenous PpETV2 and CRISPRon-mediated endogenous NPAS4L. Note that SU5402 abolishes all endogenous SCL/TAL1 expression outside the exogenous overexpression territory. Scale bar: 1 mm.

These data suggested that in ancestral amniotes, as in extant teleost fish, both NPAS4L and ETV2 were expressed in ventral mesoderm cells and were involved in blood and endothelial differentiation, with NPAS4L functioning as the major inducer for both lineages and ETV2 as a potent inducer for the endothelial lineage. However, from the perspective of phylogenetic conservation, neither can be considered the hemangioblast master regulator in amniotic vertebrates. The concept of “master regulator” was coined to describe those genes that sit at the top of a lineage specification hierarchy. Pinpointing an exact step for the origin of hemangioblasts is difficult as vertebrate development is a continuous process. In mammals, Flk1+ (VEGFR2+) mesoderm cells give rise to ETV2+ hemangioblasts (Kataoka et al, 2011; Zhao & Choi, 2017). In birds, we had previously described the origin of hemangioblasts as part of ventral mesoderm precursors located in the posterior two-thirds of the primitive streak (Nakazawa et al, 2006; Alev et al, 2010). But the identity of hemangioblasts appears after gastrulation EMT (epithelial–mesenchymal transition) (Nakazawa et al, 2006; Nakaya et al, 2008; Shin et al, 2009), after smooth muscle lineage segregation from the same pool of post-EMT mesoderm cells (Shin et al, 2009) (Fig 1A), during early migration of post-EMT mesoderm cells (Weng et al, 2007; Weng & Sheng, 2014; Nagai et al, 2018) and before aggregation of individually specified hemangioblasts as blood islands (Weng et al, 2007; Sheng, 2010). To test whether hemangioblast specification is affected by gastrulation EMT and mesoderm migration, we overexpressed SNAI2 in chicken mesoderm. SNAI2 is an EMT regulator and is normally expressed in the primitive streak and a subset of nascent mesoderm cells (Fig 4A and B) that do not overlap with NPAS4L positive cells (Fig 2G), whereas another EMT transcriptional factor, ZEB2, is mainly expressed in neural ectoderm territory, not in the streak or nascent mesoderm, and weakly in later-stage blood island cells (Fig 4C and D). Overexpression of SNAI2 did not affect normal expression of SCL/TAL1 (0/10) (Fig 4E), LMO2 (0/4) or HAND2 (0/4) (Fig 4F), suggesting that cellular differentiation is regulated separately from cellular morphogenesis. This was in agreement with data from our previous explant experiments (Nakazawa et al, 2006), in which posterior primitive streak tissues could differentiate into hematopoietic cells without undergoing proper EMT or migration.

### Receptor tyrosine kinase signaling functions upstream of NAPS4L-mediated hemangioblast specification in birds, similar to ETV2-mediated hemangioblast specification in mammals

To test whether receptor tyrosine kinase-mediated signaling could regulate avian hemangioblast specification, as demonstrated in mammals, we treated early gastrulation chick embryos (stage HH2/3) with SU5402 (a potent inhibitor of receptor tyrosine kinases, including VEGRs and FGFRs). We had previously shown SU5402 treatment after HH6/7 (when hemangioblast specification is complete) could promote blood lineage differentiation among the hemangioblasts (Nakazawa et al, 2006), a role mediated through FGFR signaling. When chick embryos were treated at HH1 (before gastrulation), both streak formation and gastrulation were severely affected (data not shown). However, when embryos were treated at early mid-gastrulation stages (HH2/3), effect on mesoderm formation was mild. Interestingly, SU5402 treatment at HH2/3 completely abolished hemangioblast marker expression, including NPAS4L (Fig 4G and H), SCL/TAL1 (Fig 4I and J), and LMO2 (Fig 4M–P), whereas smooth muscle cell marker (HAND2) expression was strongly increased (Fig 4K and L), suggesting that VEGFR-mediated signaling plays a role in hemangioblast specification in birds, in agreement with similar observations in mammals. In the presence of SU5402, however, ETV2 (6/7) retained its ability to induce endogenous chicken LMO2 expression (Fig 4M and N), but was still unable to induce endogenous SCL/TAL1 (0/5) or endogenous NPAS4L (0/5). This was partly in agreement with the observation that in the mammalian model, exogenous ETV2 could rescue *FLK1*-mutant phenotypes (Rasmussen et al, 2013). With SU5402, CRISPRon-mediated ectopic expression of endogenous NPAS4L (as we had previously reported [Weng et al, 2020]) retained its ability to induce SCL/TAL1 and LMO2 (data not shown). Either ETV2 or NPAS4L alone could induce endogenous LMO2 expression, and only NPAS4L could induce SCL/TAL1 expression (Fig 3) (Weng et al, 2020). Combined expression of exogenous ETV2 and CRISPRon-mediated endogenous NPAS4L did not have synergistic effect on either SCL/TAL1 or LMO2 induction (Fig 4O–R) (i.e., SCL/TAL1 induction by NPAS4L; LMO2 induction by both ETV2 and NPAS4L), which was not affected in the presence of SU5402 (compare Fig 4O with Fig 4P and Fig 4Q with Fig 4R).

Taken together, in this work, we showed that ancestral amniotes had both *ETV2* and *NPAS4L* genes in their genomes and that the *ETV2* gene was lost in the reptilian lineage leading to modern birds. A separate event led to the loss of *NPAS4L* gene in ancestral mammals. In reptilian species retaining both genes in their genome, ETV2 promotes endothelial lineage specification, similar to the scenario in anamniotes. Receptor tyrosine kinase-mediated hemangioblast specification may act by promoting chromatin accessibility of phylogenetically conserved downstream transcriptional regulators (e.g., SCL/TAL1 and LMO2) through NPAS4L in birds and ETV2 in mammals. A recent epigenetic study showed that ETV2 binding to its target sequence in mouse *SCL/TAL1* enhancer could be distinguished from ETV2-mediated activation of *SCL/TAL1* transcription (Steimle et al, 2023), suggesting that ETV2 may function as a pioneer factor in hematopoietic and vascular development (Gong et al, 2022; Steimle et al, 2023). Although NPAS4L has not been shown to bind to closed chromatin or function as a pioneer factor (Marass et al, 2019), its paralogue, NPAS4, can regulate neuronal-specific gene expression in an activity-dependent manner and through differential cofactor recruitment by its heterodimeric partner ARNT2 (Sharma et al, 2019). These data indicate that both NPAS4L and ETV2 may regulate lineage specification via uncoupling of target DNA binding-site occupancy and target gene transactivation, permitting additional steps of molecular regulation for fine-tuned control of hemangioblast differentiation. It is also worth noting that our current study focuses on primitive hematopoietic development and its associated endothelial lineage specification, taking place before the establishment of circulation. Involvement of NPAS4L in definitive hematopoiesis, especially with regard to the generation of hematopoietic stem cells from the dorsal aorta endothelium, awaits further study.

## Materials and Methods

### Phylogenomics analysis and single cell RNAseq data analysis

To determine whether the *Etv2* and *Fli1b* genes were lost in extant birds, we annotated these two genes and three neighboring genes (*Cox6b1*, *Rbm42*, *Haus5*) on 363 bird genomes from the B10K project ([Feng et al, 2020] for list of bird species). As reference sets, we collected protein sequences of these genes from the following five species: *Homo sapiens*, *Lacerta agilis*, *Sphaerodactylus townsendi*, *Gallus gallus*, and *Taeniopygia guttata*. Two homologous ETS gene pairs (*Ets1-Fli1* and *Ets2-Erg*) of *Etv2*-*Fli1b* were also included in the reference set to avoid annotation errors caused by similarity. The reference protein sequences were aligned to the avian genomes by Exonerate (v2.4.1) for gene annotation. To filter out low-quality annotation outcomes, all predicted gene models were translated into protein sequences and then aligned with the reference protein sequence with Muscle (v3.8.1551). Gene models with fewer than 30 amino acids and less than 40% identity to the reference protein were removed. For the loci with multiple annotated gene models, we only kept the one with the highest identity to the reference protein. *Etv2* and *Fli1b* genes were not found in any of the B10K genomes, but neighboring genes were annotated in some avian genomes. To minimize false loss caused by poor assembly quality, we further checked the NCBI gene set database and noted no *Etv2* and *Fli1b* genes in any birds, but we did locate neighboring genes in some birds. We chose birds with high-quality genomes from different clades to demonstrate the presence or absence of *Etv2* and *Fli1b* and three neighboring genes.

Raw reads for chicken early embryogenesis scRNAseq were obtained from GEO NCBI (GSE181577 [Williams et al, 2022] and GSE223189 [Rito et al, 2023 *Preprint*]) and processed with cellranger-7.0.1 using galGal6 (GCF_000002315.6) as a reference genome assembly. Next expression matrices were filtered (min cell size 500) and normalized by Pagoda2 (v1.0.11; number of top overrepresented genes 2,000, number of PCs 50). Integration was conducted with Conos (v1.5.0) (Barkas et al, 2019) using k.self = 10, ncomps = 30, and n.odgenes = 2000. Human gastrulation data were used for annotation inference (Tyser et al, 2021). Cell clusters were identified with the Leiden algorithm.

### Embryology, RNA in situ hybridization, and expression constructs

Fertilized hens’ eggs were obtained from a local farm in Aso. Madagascar ground gecko colony was maintained in RIKEN BDR. Embryological analysis of chicken embryos followed standard protocols as previously described (Alev et al, 2013; Weng et al, 2020). Intra-uterine gecko embryo collection was described previously (Kajikawa et al, 2020), and RNA in situ analysis with gecko embryos followed the chicken protocol (Alev et al, 2013).

Madagascar ground gecko (*Paroedora picta*) *NPAS4L* (comp58593_c0_seq1) was identified by tblastx on Reptiliomix (https://transcriptome.riken.jp/reptiliomix/) with green anole (*Anolis carolinensis*) *NPAS4* (XM_008104927) (this gene is the *NPAS4L* ortholog but is incorrectly annotated as *NPAS4*) and confirmed by NCBI blast. A 1,068 bp fragment corresponding to nucleotides 798–1,865 of comp58593_c0_seq1 was amplified and used for RNA in situ hybridization. Sequence (comp295360_c0_seq3) containing Madagascar ground gecko *ETV2* was identified by tblastx on Reptiliomix with green anole *ETV2* (XM_008120938) and verified by NCBI blast. A 567 bp fragment corresponding to nucleotides 526–1,092 of comp295360_c0_seq3 was selected for RNA in situ hybridization.

Application of CRISPRa (also known as CRISPRon) technology in avian embryos was described previously (Lizio et al, 2017; Weng et al, 2020). Four sgRNA sequences (GCCCATGTCTGAGGGAGAGA, TCTCTCCCTCAGACATGGGC, GGAAACCCAGAGGTGCCCAG, CTGGGCACCTCTGGGTTTCC) within the 400 bp region before the transcription start site of chicken p68ETS1 (https://fantom.gsc.riken.jp/zenbu; galGal5::chr24:857052..961686+) were cloned into pAC154 dual-dCas9VP160-sgExpression vector (#48240; Addgene) for ETS1 CRISPRa. Full-length coding regions of gecko ETV2 (comp295360_c0_seq3), mouse ETV2 (NM_007959), chicken p68ETS1 (XM_015297968), and chicken p54ETS1 (XM_040652038) were inserted into pCAGGS-2AGFP (Weng & Sheng, 2014) expression vector for overexpression in chick embryos. Expression constructs for p68ETS1 and p54ETS1 were generated by cloning full-length sequences (154–1,608 of XM_015297969.2 and 176–1,548 of X13027.1, respectively) into pCAGGS-2A-eGFP vector described previously (Weng & Sheng, 2014). cDNA sequence for chicken SNAI2 (XM_040664699.1; coding region 3,450–4,256) was cloned into pCAGGS-2A-eGFP for making SNAI2 expression construct. Plasmid constructs were diluted before electroporation to a final concentration of 1 mg/ml per construct for electroporation in Pannett-Compton solution with glycerol (final concentration 10%) and Fast Green (#061-00031; Wako; final concentration 0.1%).

## Supplementary Material

Reviewer comments
